# Primary testicular neuroendocrine tumor with retroperitoneal lymph nodes metastasis: a case report

**DOI:** 10.3389/fonc.2026.1766386

**Published:** 2026-05-08

**Authors:** Ming Zhang, Qian Wang, Fahimullah Hamid, Jiehao Wu, Jiaxue Han, Pengfei Shen

**Affiliations:** Department of Urology, Institute of Urology, West China Hospital, Sichuan University, Chengdu, Sichuan, China

**Keywords:** case report, metastasis, orchiectomy, surufatinib, testicular neuroendocrine tumor

## Abstract

Testicular neuroendocrine tumor (TNET) is an exceedingly rare malignancy with variable biological behavior. We report a 49-year-old male presenting with painless right testicular enlargement. Primary TNET (G1) was diagnosed after extensive pathological sampling and the clinical exclusion of extra-testicular primary sites via contrast-enhanced CT. Following radical orchiectomy, the patient underwent laparoscopic retroperitoneal lymph node dissection (RPLND), which confirmed metastatic involvement (7/13 nodes). Although severe intraoperative adhesions to the great vessels were encountered, a thorough dissection was performed, and postoperative imaging showed no measurable macroscopic residual disease, consistent with a clinical R0 resection. Based on a multidisciplinary team (MDT) assessment of the high risk for subclinical microscopic residuals, adjuvant therapy with surufatinib, a multi-targeted TKI, was initiated. A complete response (CR) was achieved eight months postoperatively, defined by the disappearance of all target lesions on follow-up contrast-enhanced CT according to RECIST 1.1 criteria. The patient remains disease-free at the one-year follow-up, suggesting that targeted therapy may be a promising and effective option for metastatic G1 TNET following surgical debulking.

## Introduction

Testicular tumor is one of the most common malignant tumors of the male genitourinary system. Although it accounts for only 1% of all adult cancers, its incidence is higher in young men aged 15 to 35 years (1–3). Germ cell tumors (GCTs) are the most common type, accounting for approximately 90-95% of cases, which include seminomas and non-seminomatous germ cell tumors (NSGCTs) (4). Testicular neuroendocrine tumors (TNET) are a rare subtype, typically characterized by high invasiveness and poor prognosis (5). The immunophenotype of testicular neuroendocrine tumor often shows positivity for Synaptophysin (Syn), Chromogranin A (CgA), and CD56, distinguishing it from other types of testicular tumors (6).

In the metastatic process of testicular tumor, retroperitoneal lymph nodes are a common site of metastasis, especially in non-seminomatous and neuroendocrine subtypes of testicular tumor (7). Studies have shown that retroperitoneal lymph nodes are the “first station” for testicular tumor metastasis, particularly during lymphatic spread (8). Right-sided testicular tumors are more likely to metastasize to the lymph nodes between the abdominal aorta and inferior vena cava due to anatomical features (9). However, it is important to note that some highly invasive neuroendocrine testicular tumors may exhibit earlier hematogenous metastasis rather than solely lymphatic spread (10). Despite this, retroperitoneal lymph node metastasis remains a key factor in determining the prognosis of testicular tumor (11). Most patients may be asymptomatic in the early stages, and as the disease progresses, retroperitoneal lymph node metastasis often presents with abdominal discomfort and bilateral lower limb edema (12). For testicular tumor patients with retroperitoneal lymph node metastasis, timely surgical removal of the primary tumor and metastatic lymph nodes, combined with chemotherapy or targeted therapy, is crucial for improving survival (13).

The management of germ cell tumors is characterized by prolonged treatment courses and complex therapeutic regimens, which require close multidisciplinary collaboration. Throughout the treatment process, coordinated assessment by surgeons, medical oncologists, and radiologists is essential. An integrated, multidisciplinary approach has the potential to further improve patient survival. We present the case of a 49-year-old male patient diagnosed with testicular neuroendocrine tumor with retroperitoneal lymph node metastasis. After comprehensive treatment by a multidisciplinary team, including primary tumor resection, retroperitoneal lymph node dissection, and targeted drug therapy, significant therapeutic effects were achieved. This case provides insight into the diagnosis and treatment strategy for testicular neuroendocrine tumor with retroperitoneal lymph node metastasis, offering valuable clinical implications.

## Case presentation

A 49-year-old male presented with a painless swelling of the right testicle for over six months. He had a history of cholecystectomy and appendectomy. Physical examination revealed a hard mass in the right testicle (approximately 5 cm), with limited mobility and no obvious tenderness; inguinal lymph nodes were not palpable. Color Doppler ultrasound of the male reproductive system indicated a solid-cystic mass in the right testicle.

To exclude a potential extra-testicular primary source, extensive imaging workups were performed. Preoperative chest and abdominal enhanced CT showed enlargement of the right testicle with a transverse diameter of approximately 5.5 cm × 5.5 cm; a nodular lesion near the right abdominal aorta was identified, suggesting possible lymph node metastasis (approximately 1.5 cm in diameter). No evidence of primary malignancy in the gastrointestinal tract, lungs, or pancreas was observed. All tumor markers were negative, with beta-human chorionic gonadotropin (β-HCG) <0.6 mIU/mL, alpha-fetoprotein (AFP) 5.22 ng/mL, and D-dimer 3.07 mg/L, while other hematological parameters were within the normal range. After preoperative examination and the exclusion of surgical contraindications, right orchiectomy was performed. Intraoperatively, the right testicle showed a hard, nodular mass, approximately 5 cm in diameter, adherent to the scrotal wall. The testicle was dissected, and it showed a fish-like appearance upon incision (**Figure 1**). The histological specimen was thoroughly examined via extensive sampling. Immunohistochemical analysis confirmed the neuroendocrine nature of the tumor, with positive staining for CK (Pan), CD56, Syn, and CgA. To exclude sex cord-stromal tumors and other mimics, additional markers including Inhibin, SF-1, WT1, Vimentin, and FoxL2 were tested, all of which were negative. The Ki-67 labeling index was 1%, consistent with a grade 1 (G1) neuroendocrine tumor. No components of teratoma or germ cell tumor *in situ* were identified, supporting the diagnosis of a pure primary testicular NET. (**Figures 2A, B**).

**Figure 1:**
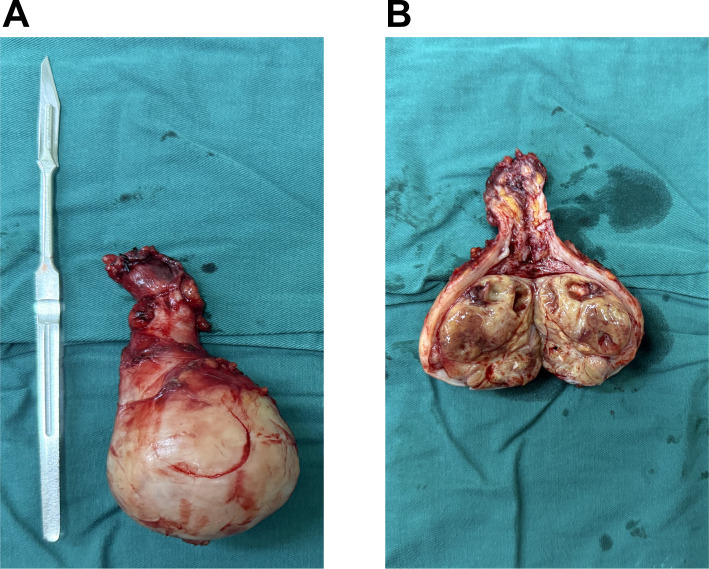
Gross specimen of the right testis. **(A)** Overall view of the specimen. **(B)** Longitudinal section of the specimen.

**Figure 2:**
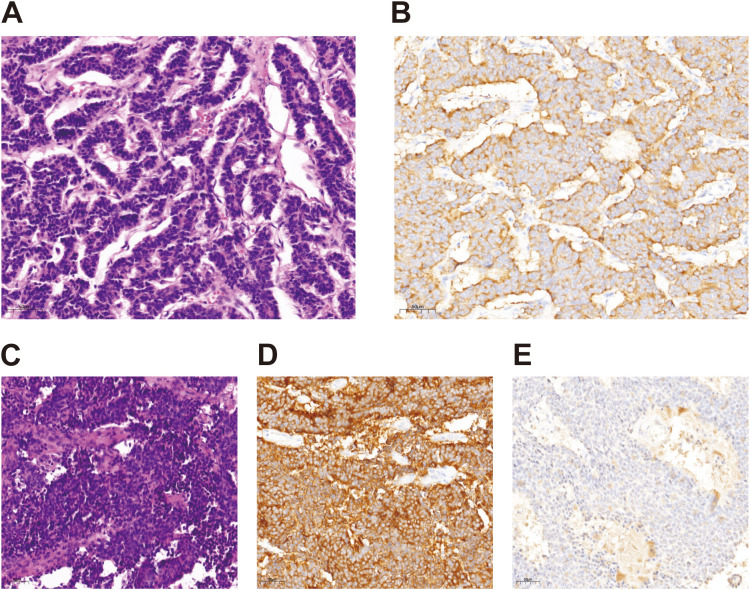
Histopathological and immunohistochemical characterization of the primary tumor and metastatic lymph nodes. **(A)** Hematoxylin and eosin (H&E) staining of the right testicular tumor showing highly differentiated neuroendocrine cells arranged in an organoid, nested pattern with characteristic nuclear chromatin. **(B)** Immunohistochemical (IHC) staining of the primary tumor demonstrating strong and diffuse cytoplasmic expression of Synaptophysin (Syn), supporting the neuroendocrine origin (200×). **(C)** H&E staining of the right retroperitoneal lymph node showing metastatic involvement with morphological features consistent with the primary testicular tumor (200×). **(D)** Diffuse and intense cytoplasmic positivity for Synaptophysin (Syn) in the metastatic lymph node tissue (200×). **(E)** Immunohistochemical staining for Chromogranin A (CgA) in the metastatic lymph node, showing focal positive immunoreactivity (200×).

Two months after the initial surgery, which allowed for sufficient physical recovery and multidisciplinary team (MDT) discussion, an 18F-fluorodeoxyglucose (18F-FDG) positron emission tomography-computed tomography (PET/CT) scan revealed elevated glucose metabolism in the retroperitoneal lymph nodes near the right abdominal aorta (**Figure 3**), with a maximum standardized uptake value (SUVmax) of 3.75. The findings suggested nodal metastasis, with the largest lesion measuring approximately 2 cm. Following a MDT discussion, the patient was recommended for retroperitoneal lymph node dissection (RPLND). During the laparoscopic RPLND, enlarged lymph nodes (approximately 2 × 2 cm) were identified near the abdominal aorta and inferior vena cava. Although severe adhesions to the aortic and venous walls were encountered, the operating surgeon performed a meticulous dissection, and the metastatic territory was thoroughly cleared. Postoperative contrast-enhanced CT showed only postoperative changes with no definite evidence of macroscopic residual masses, consistent with a clinical R0 resection. Pathological examination of the excised tissue revealed metastasis from a grade 1 neuroendocrine tumor in the right retroperitoneal lymph nodes (7/13). Immunohistochemistry results were positive for CK (Pan), Syn, CgA, CD56, and Ki-67 (1-2%) (**Figures 2C–E**).

**Figure 3:**
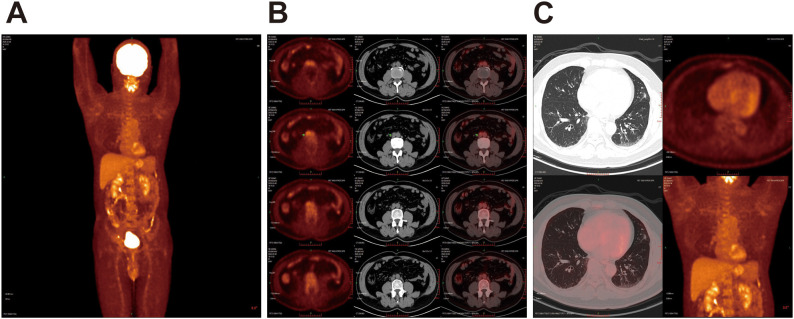
^18^F-FDG PET/CT imaging after radical orchiectomy. **(A)** The ^18^F-FDG PET/CT scan revealed the systemic tumor burden two months after right orchiectomy. **(B)** The ^18^F-FDG PET/CT scan revealed that glucose metabolism was elevated in the retroperitoneal lymph nodes near the right abdominal aorta. **(C)** The ^18^F-FDG PET/CT scan showed no lesion of increased glucose metabolism in both lungs.

Postoperatively, the patient continued follow-up in the department of oncology and initiated oral surufatinib at 300 mg daily. One month after the RPLND, a 68Ga-labeled somatostatin analogue (68Ga-DOTATATE) PET/CT was performed to evaluate the somatostatin receptor 2 (SSTR2) status and systemic tumor burden. The scan showed no definite evidence of SSTR2-positive recurrence or metastasis (**Figure 4**). During surufatinib treatment, the patient exhibited a favorable safety profile with no significant treatment-emergent adverse events reported, demonstrating excellent drug tolerance. Eight months postoperatively, follow-up contrast-enhanced CT demonstrated the complete disappearance of the residual para-aortic lymph nodes. According to the Response Evaluation Criteria in Solid Tumors (RECIST) version 1.1, the patient achieved a complete response (CR), with no evidence of disease recurrence or new metastasis observed during over one-year follow-up period.

**Figure 4:**
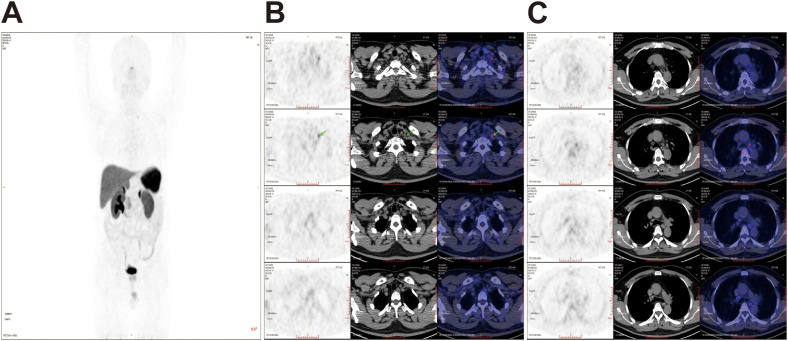
^68^Ga-DOTATATE PET/CT assessment following RPLND. **(A)** The ^68^Ga-DOTATATE PET/CT scan revealed the systemic tumor burden one months after postoperative changes in the right abdominal wall, inguinal region, para-aortic area, and mesenteric territory. **(B)** The ^68^Ga-DOTATATE PET/CT scan revealed postoperative changes in the right abdominal wall, inguinal region, para-aortic area, and mesenteric territory. **(C)** The ^68^Ga-DOTATATE PET/CT scan showed no abnormal ^68^Ga-DOTATATE avidity in both lungs.

## Discussion

Testicular neuroendocrine tumor is a rare entity with variable biological behavior, accounting for only 0.2% of all testicular tumors (5). Its rarity and nonspecific clinical presentation, which often overlaps with more common germ cell tumors, pose significant diagnostic pitfalls. As observed in our case and supported by a meta-analysis of 132 TNET cases, most patients present with a painless mass and normal serum tumor markers (AFP, β-HCG, and LDH) (14). The primary diagnostic challenge lies in distinguishing “pure” TNET from metastatic NET or TNET arising within a teratoma, the latter of which may follow a different clinical course (15–17). In our patient, the integrated use of medical imaging and a specific immunohistochemical panel was not merely supportive but essential for diagnostic exclusion. Specifically, PET/CT was instrumental in ruling out an extra-testicular primary origin, while the negative expression of germ cell and sex cord-stromal markers confirmed the “pure” nature of the TNET. This multimodal diagnostic approach is critical for navigating the pitfalls associated with such a rare and poorly defined entity.

While radical orchiectomy remains the standard for controlling the primary lesion and providing definitive staging, the management of metastatic disease is far less standardized. The role and limits of RPLND in TNET deserve careful consideration. In conventional germ cell tumors, the lymphatic spread follows a well-established anatomical drainage to the para-aortic and interaortocaval nodes (18, 19). However, for TNET, the therapeutic benefit of RPLND is less clear, especially for G1 lesions. In this case, although the metastatic nodal mass was inextricably adhered to the great vessels, a meticulous dissection was performed to clear the metastatic territory. Postoperative imaging identified no measurable macroscopic residual masses, consistent with a clinical R0 resection. However, this surgical experience underscores a critical clinical uncertainty: the presence of severe intraoperative adhesions suggests a high risk of microscopic or subclinical residual disease, even when radiologically negative.

Management of metastatic TNET requires a multidisciplinary approach, with a comprehensive treatment strategy being essential (20). While radical orchiectomy, a standard treatment, and RPLND effectively reduce the visible tumor burden and provide a definitive pathological basis, the potential for subclinical recurrence remains a challenge. The management of metastatic testicular NETs with gross residual disease remains a clinical challenge, and the selection of adjuvant therapy requires careful consideration of tumor biology. Current systemic and local adjuvant options include observation, somatostatin analogs (SSAs), targeted therapies, radiotherapy, systemic chemotherapy. In this case, adjuvant chemotherapy and radiotherapy were not pursued based on a MDT consensus. Conventional cytotoxic chemotherapy (e.g., platinum-based regimens) is typically reserved for high-grade neuroendocrine carcinomas (G3) with rapid proliferation. In contrast, well-differentiated G1 NETs, such as the one in our patient (Ki-67 index of 1%), are inherently resistant to chemotherapy due to their slow mitotic rate. Similarly, the role of radiotherapy for gross residual disease in the aortocaval region is poorly defined for low-grade NETs. The low proliferative activity of G1 cells translates to limited radiosensitivity, and the risks of radiation-induced morbidity often outweigh the marginal local control benefits.

The selection of surufatinib as the systemic treatment was justified by both current clinical guidelines and its unique pharmacological profile (21). Surufatinib is an oral TKI that simultaneously targets vascular endothelial growth factor receptors (VEGFR 1, 2, and 3), fibroblast growth factor receptor 1 (FGFR1), and colony-stimulating factor 1 receptor (CSF-1R). Unlike many other TKIs, its triple-target mechanism exerts potent anti-angiogenic effects while modulating the tumor immune microenvironment by inhibiting tumor-associated macrophages. Based on the robust evidence from the SANET-ep and SANET-p phase III trials, surufatinib has been officially recommended as a standard systemic therapy for G1/G2 neuroendocrine tumors (22–24). Given the severe intraoperative adhesions and the associated risk of microscopic residuals, this systemic targeted approach offered a more comprehensive therapeutic benefit compared to localized measures. The effectiveness of this strategy was retrospectively validated by the achievement of a sustained CR according to RECIST 1.1 criteria, with the complete disappearance of all postoperative anatomical shadows at the para-aortic site.

In prognostic evaluation of testicular neuroendocrine tumor, the Ki-67 proliferative index and tumor differentiation are key prognostic factors (6). Studies have shown that lower Ki-67 indexes and better tumor differentiation correlate with better prognosis (25). In this case, the patient’s low Ki-67 index and good tumor differentiation suggest a relatively favorable prognosis. Although the presence of retroperitoneal lymph node metastasis increases the complexity and difficulty of treatment, combined surgery and targeted therapy effectively controlled the disease. Currently, the patient remains disease-free with no new metastasis or disease activity, suggesting a positive treatment outcome. However, long-term follow-up is still required to assess potential recurrence or metastatic lesions.

This case provides valuable insights into the treatment of testicular neuroendocrine tumor. Surgical resection played an important role in controlling the primary tumor, confirming the diagnosis, and reducing tumor burden, while targeted therapy effectively controlled metastatic disease. Although there is no standardized treatment protocol for testicular neuroendocrine tumor (26), this case suggests that individualized treatment, particularly combining surgery with targeted drugs, may offer a potential therapeutic strategy for similar cases. However, the long-term efficacy of combined surgery and surufatinib remains to be established, as this report is based on a single case with a relatively short follow-up period. As awareness of this rare cancer grows, more standardized and personalized treatment strategies may be developed to optimize outcomes and prognosis for these patients.

## Conclusion

Managing metastatic G1 TNET is challenging given its limited responsiveness to conventional adjuvant treatments. Our study suggests that the integration of surgical debulking and adjuvant targeted therapy with surufatinib provides an effective means of achieving a sustained complete response. While this case offers a promising therapeutic strategy for such rare malignancies, extended follow-up is necessary to validate the long-term clinical outcomes.

## Data Availability

The original contributions presented in the study are included in the article/supplementary material. Further inquiries can be directed to the corresponding author.
